# Binocular combination of stimulus orientation

**DOI:** 10.1098/rsos.160534

**Published:** 2016-11-16

**Authors:** O. Yehezkel, J. Ding, A. Sterkin, U. Polat, D. M. Levi

**Affiliations:** 1 School of Optometry and Helen Wills Neuroscience Institute, UC Berkeley, Berkeley, CA 94720, USA; 2 Goldschleger Eye Research Institute, Sackler Faculty of Medicine, Tel Aviv University, Sheba Medical Center, Tel Hashomer 52621, Israel; 3 Faculty of Life Sciences, Optometry and Vision Sciences, Bar-Ilan University, Ramat-Gan, Israel

**Keywords:** binocular combination, orientation, contrast, DSKL model, interocular suppression, interocular enhancement

## Abstract

When two sine waves that differ slightly in orientation are presented to the two eyes separately, a single cyclopean sine wave is perceived. However, it is unclear how the brain calculates its orientation. Here, we used a signal detection rating method to estimate the perceived orientation when the two eyes were presented with Gabor patches that differed in both orientation and contrast. We found a nearly linear combination of orientation when both targets had the same contrast. However, the binocular percept shifted away from the linear prediction towards the orientation with the higher contrast, depending on both the base contrast and the contrast ratio. We found that stimuli that differ slightly in orientation are combined into a single percept, similarly for monocular and binocular presentation, with a bias that depends on the interocular contrast ratio. Our results are well fitted by gain-control models, and are consistent with a previous study that favoured the DSKL model that successfully predicts binocular phase and contrast combination and binocular contrast discrimination. In this model, the departures from linearity may be explained on the basis of mutual suppression and mutual enhancement, both of which are stronger under dichoptic than monocular conditions.

## 1. Introduction

The human visual system combines slightly different visual inputs from the two eyes into a single coherent cyclopean percept in which the two inputs are ‘fused’. Binocular combination has been studied for almost two centuries [1], using both neurophysiological and behavioural approaches involving a wide variety of stimuli and tasks [2–24]. The simplest theory of binocular combination is linear summation, in which the combined fused image is the linear sum of the images presented to each eye. However, the common behavioural finding is that performance (sensitivity) benefits from binocular viewing when compared with monocular only by about the square root of two (approx. 40%) for detection tasks, and the benefit is even lower for discrimination tasks (for a review, see [25], with numerous models offering explanations for this nonlinearity [25], such as gain-control theory [6,16,26]). Binocular combination is also affected by the physical properties of the stimuli, such as stimulus energy, becoming more linear at shorter durations and lower contrasts [16,17,27].

Much less is known about the mechanism of binocular combination. To date, this question has been approached by measuring either the perceived phase or contrast of cyclopean gratings [6,7,14]. Surprisingly, binocular combination of orientation has received little attention. Understanding how inputs are combined when they differ slightly in orientation is important, because the stimulus orientations in the two eyes may be slightly different because of the viewing geometry or slight eye torsion. In particular, surfaces that are slanted about a horizontal axis create orientation disparities between corresponding vertical lines [28].

Classical receptive fields of simple cells are closely matched by Gabor signals [29,30], similarly for behavioural and single-cell-level measurements [31]. However, in a substantial proportion of V1 neurons, the preferred orientations may differ, with interocular differences of approximately ±15 degrees in the preferred orientations of neurons found in cat striate cortex [32]. In the macaque, roughly one-third of V1 neurons differed significantly in the preferred orientations of the two eyes [33].

Humans perceive a fused orientation when the stimuli to the two eyes differ in orientation, with long horizontal lines differing in orientation between the two eyes by approximately 5 degrees fused *without* rotational eye movements [34]. Furthermore, briefly exposed short vertical lines appeared fused for orientation difference of up to approximately 30 degrees [35]. Here, we presented brief Gabor signals to the two eyes that differed in orientation by 10–20 degrees from vertical, and measured the perceived cyclopean orientation. Thereby, we directly examined the extent of binocular combination of orientation between receptive fields activated by inputs from the two eyes that are analysed by the same putative processing channel [36–39].

Interestingly, if two gratings that differ slightly in orientation (within 15–20 degrees) are presented briefly to one eye at the same time, they also appear fused [40–42]. Campbell *et al*. [40] suggest that this occurs when the two gratings activate mainly one set of orientation selective neurons. Therefore, for comparison, we also examined how Gabor signals differing in orientation were combined monocularly.

## 2. Material and methods

### 2.1. Observers

Four naive observers participated in both the dichoptic and monocular experiments (age range 18–27, three females), with normal or corrected-to-normal visual acuity, unaware of the purpose of the study. Full optometric refractions were performed, and refractive errors (including the astigmatic errors) were fully corrected. Each observer signed an informed consent form approved by the local institutional review board.

### 2.2. Apparatus

The experiments were controlled by a PC, and the stimuli were displayed as a grey-level modulation on a Philips 107P colour monitor, 100 Hz refresh rate. The mean display luminance was 40 cd m^−2^ in an otherwise dark environment. Screen resolution was 1024 × 768 pixels; gamma correction was applied. The stimuli were viewed from a distance of 150 cm.

### 2.3. Stimuli

Stimuli were localized grey-level gratings (Gabor patches, GPs) with a spatial frequency of three cycles per degree (wavelength, *λ*), equal distribution (STD, *σ*, 0.23 degrees, allowing minimum two cycles in the GP). Stimuli were presented foveally using stereo goggles (crystal eyes 3, StereoGraphycs). Cross-talk of our goggles was minimal (2.2% at the highest contrast level, determined by measuring the luminance of a patch shown to each eye through the other eye's shutter using a photometer), and was invisible to observers at the brief exposure duration.

**Figure 1.**
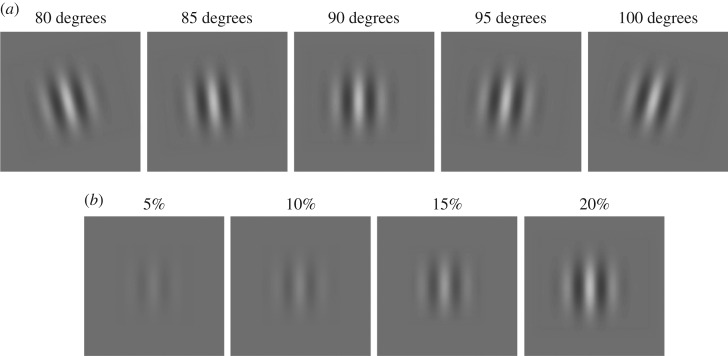
Stimuli. (*a*) Five tested orientations, at a representative contrast of 20%. (*b*) Four contrast levels that were combined with a representative base contrast of 20%, in order to create all possible interocular contrast ratios per orientation combination (table 1).

Orientations with a difference of 10, 15 and 20 degrees were combined, resulting in a total of eight combinations: 80–95, 80–100, 85–95, 85–100, 95–80, 95–85, 100–80 and 100–85 (figure 1*a*). We tested this limited set of orientation differences (up to 20 degrees) in order to avoid conditions where rivalry (either monocular or binocular) occurs. For each orientation combination, one eye (e.g. the right eye) was stimulated with a fixed higher contrast or base contrast (e.g. 20%), whereas the fellow eye was presented with one of the four contrast possibilities: 5%, 10%, 15% or 20% (figure 1*b*). This was repeated with the same base contrast (20%) in the other eye (e.g. the left eye) and the varying contrast in the fellow eye. Note that in this scheme, contrast ratio = 1 is presented twice, resulting in eight interocular contrast ratios per orientation combination (0.25, 0.5, 0.75, 1, 1, 1.33, 2 and 4; table 1). We tested four base contrasts (10%, 20%, 40% and 60%). For each base contrast, five baseline control conditions were also tested when interocular contrast ratios = 0 or *∞*, i.e. only base contrast was presented to one eye, and the other eye just viewed a blank with grey background (80, 85, 90, 95 and 100 degrees; figure 1*a*). In addition, for each base contrast and contrast ratio, the monocular control conditions were also tested when the two gratings were presented in the same eye (monocular orientation combination). During monocular presentation, the other eye was presented with a blank screen at the mean contrast level.

**Table 1.** A sample orientation-ratios matrix. The contrasts (peak-to-peak Michelson contrast, in %) used for the eight possible contrast ratios when a representative orientation combination of 80 and 100 degree GPs were presented to the left (L) and right (R) eyes, respectively. Base contrast = 20%. Note that contrast ratio = 1 is presented twice.

	base contrast 20% right eye				base contrast 20% left eye			
orientation difference 20 degrees. L (80°)/R (100°)	0.25	0.5	0.75	1	1	1.33	2	4
L (%)	5	10	15	20	20	20	20	20
R (%)	20	20	20	20	20	15	10	5

### 2.4. Visual task

The task was to rate the perceived orientation as one of 5 options, corresponding to 80, 85, 90, 95 and 100 degrees, by pressing a computer keyboard button immediately after making their decision, using the dominant hand. An additional (sixth) option was reserved for the ‘cannot decide’ response (with 0% for this response in three out of four observers and up to 5% in observer D.V.; data not shown). Each trial was preceded by a fixation circle (approx. 0.3 degrees) at the centre of the display until the observer signalled their readiness using the keyboard space buttons. Observers were instructed to begin a trial only given a clear perception of a single fixation circle to insure interocular alignment. Then, a stimulus was presented for 80 ms. No feedback was provided.

### 2.5. Orientation tuning

Each of the orientation combinations was tested both monocularly and dichoptically. There were 32 conditions (eight orientation differences × four contrast ratios). In a given run of 192 trials, we intermixed 16 conditions (all eight orientation differences and two contrast ratios—12 trials per condition). In the monocular trials, the fellow eye was presented with a mean isoluminance background, with observers unaware of the origin of stimulation. Each run was repeated four times per eye receiving the base contrast yielding 48 measurements per condition. The same condition was repeated with the base contrast switched to the other eye, yielding another 48 measurements (combining the two eyes resulted in 96 trials per condition). The total number of trials for each contrast base was 3072, yielding 12 288 trials for the four base contrasts altogether. The monocular control conditions were tested in additional 12 288 trials, using the same protocol (the monocular and dichoptic trials were not interleaved). The baseline control conditions were tested in additional 960 trials, using runs of 10 blocks of 12 trials each, repeated eight times, yielding 96 measurements per condition. Overall, a total of 25 536 trials were performed by each subject, which took approximately 40–50 h per subject to complete the experiment. The first additional run of the baseline control served as ‘preview’ training and was not included in the analysis. Data were obtained for each observer and then merged for the group analysis.

### 2.6. Data analysis

We used a rating scale signal detection analysis [43] to obtain estimates of perceived orientation for each observer and condition, using Matlab. Briefly, a stimulus–response matrix was obtained for each base contrast and orientation combination. Table 2 shows an example matrix when base contrast = 20% and the GPs with 80 and 100 degree orientation were present to the left and right eyes, respectively. To eliminate possible biases (e.g. response bias and binocular bias), for each observer, the response frequencies were pooled over the two directions of rotation and the two eyes. The signal detection theory (SDT) model was used to fit the final stimulus–response matrix. The model contains nine Gaussian distributions, differing in their mean values but with the same standard deviation, corresponding to the binocular-combined orientations with nine interocular contrast ratios. The model has a total of 14 parameters, four criteria for five response options, nine mean values of the Gaussian distributions to give the perceived orientations and their standard deviations. The mean values of the first and the last Gaussian distributions were fixed to be 80 and 100 degrees, respectively, corresponding to the control conditions when interocular contrast ratio = 0 and *∞*.

**Table 2.** A sample stimulus–response matrix. The frequencies of orientation responses of 80, 85, 90, 95 and 100 degrees when 80 and 100 degree GPs were presented to the left (L) and right (R) eyes, respectively. The frequencies were summed over the two directions of rotation and the two eyes. The eight interocular contrast ratios L/R = 0, 0.25, 0.5, 0.75, 1,1, 1.33, 2, 4, or ∞, the base contrast = 20% and stimulus duration = 80 ms. (Observer = O.E.). Note that the frequency of orientation responses is double for contrast ratio = 1 because ratio 1 was run twice.

	frequencies of orientation responses				
L(100)/R(80) contrast ratio	80 degrees	85 degrees	90 degrees	95 degrees	100 degrees
0	91	95	4	2	0
0.25	73	105	13	1	0
0.5	34	124	32	2	0
0.75	19	80	85	7	1
1	3	96	186	96	3
1.33	1	7	85	80	19
2	0	2	32	124	34
4	0	1	13	105	73
*∞*	0	2	4	95	91

Table 3 shows the parameters of the SDT model that best fit the sample stimulus–response matrix shown in table 2. The mean values of the first and the last Gaussian distributions were fixed to be 80 and 100 degrees, respectively (i.e. *µ*_1_ = 80 and *µ*_9_ = 100, the perceived orientations under control conditions when the contrast ratio = 0 and *∞*). The perceived orientation at a given test contrast ratio is given by the mean value of its Gaussian distribution. Using this method, we estimated the perceived orientations for all conditions for each observer, and averaged the results across the four observers.

**Table 3.** SDT model parameters. The reduced *χ*^2^ = 1.22; the number of degrees of freedom = 24; *µ*_1 _= 80 and *µ*_9 _= 100 were fixed. The standard errors were estimated from the standard deviation of the residuals and the Jacobean matrix at the point of best fit in parameter space.

*µ*_2_	*µ*_3_	*µ*_4_	*µ*_5_	*µ*_6_	*µ*_7_	*µ*_8_	*Cr*_1_	*Cr*_2_	*Cr*_3_	*Cr*_4_	*σ*
80.8 ± 0.8	83.2 ± 0.8	86.3 ± 0.7	90.0 ± 0.6	93.6 ± 0.7	96.8 ± 0.8	99.2 ± 0.8	79.5 ± 0.6	87.0 ± 0.5	93.0 ± 0.5	100.5 ± 0.6	4.7 ± 0.2

### 2.7. Contrast detection threshold

Binocular and monocular presentations with a GP (90 degrees orientation) were used to measure contrast detection thresholds per observer. Monocular and dichoptic trials were intermixed, whereas in the monocular trials, the fellow eye was presented with a mean luminance background, with observers unaware of the origin of stimulation. A one-up three-down staircase procedure was used to estimate thresholds. Data were obtained for each observer and then merged for the group. The averaged thresholds were 5.0 ± 0.52% for monocular contrast detection, and 3.2 ± 1.80% for binocular contrast detection—a binocular summation ratio of approximately 1.56—well within the range reported in previous studies [25].

### 2.8. Modelling

When the two eyes were presented with two GPs with slightly different orientations, a single fused GP was perceived. In order to better understand the mechanisms underlying binocular combination of orientation, we tested several models.

The simplest model is *linear vector summation.* If a GP of one orientation is represented by a vector ***GP***(*m*, *θ*), with the vector length representing the GP's contrast *m*, and the vector angle representing the orientation angle *θ*, the linear summation model assumes that the fused GP is the linear vector summation of the two monocular GP vectors. Note that this vector summation is not the real mathematical summation of two GPs with different orientations. Let ***GP***(*m*_1_, *θ*_1_) and ***GP***(*m*_2_, *θ*_2_) be two GPs presented to the two eyes, respectively. The fused GP vector is given by $\text{GP}\text{(}\hat{m},\hat{\theta }\text{) = }\text{GP}(m_{1},\theta_{1})+\text{GP}(m_{2},\theta_{2})$. The perceived orientation is given by

2.1 $$\hat{\theta }=\tan^{-1}\left(\frac{m_{2}-m_{1}}{m_{2}+m_{1}}\tan\frac{\theta_{2}-\theta_{1}}{2}\right)+\frac{\theta_{2}+\theta_{1}}{2}.$$

Ding & Sperling [6] proposed an interocular gain-control model to explain binocular phase combination. Later, the model was modified by adding interocular gain-enhancement to explain both binocular phase and contrast combination (DSKL model—[7]). Ding *et al*. [7] compared five nested models to predict both phase and contrast binocular combination. The first nested model was a contrast-weighted summation model, a simplified Ding–Sperling model with the gain-control threshold = 0, which can explain binocular phase combination [6,7], but failed to predict both phase and contrast in a binocularly combined sine wave [7]. The second and third nested models were the Ding–Sperling models with symmetric (the second) or asymmetric (the third) double-layer interocular gain-controls. The fourth nested model added interocular gain-enhancement to the third model, and the full model (the fifth nested model) added interocular gain-control of gain enhancement to the fourth model.

In this study, we set out to test these five nested models for orientation combination. However, it is very difficult to obtain reliable data for perceived orientation when the stimulus contrast is near threshold. Thus, in the model fitting, the gain-control thresholds were not significant and we therefore fixed the gain-control threshold and the threshold for gain-control of gain-control to be 0 for all five nested models. This makes the first three nested models identical. However, mutual enhancement had to be assumed to account for the decreasing mutual suppression when the base contrast increases. Therefore, we tested only three nested models in this study.

*Model 1: contrast-weighted summation model (simplified Ding–Sperling model)*. The Ding–Sperling model can be simplified to be a contrast-weighted summation model when the gain-control threshold = 0. The fused GP vector is given by

2.2 $$\text{GP}(\hat{m},\hat{\theta })=\frac{m_{1}^{\gamma }}{m_{1}^{\gamma }+m_{2}^{\gamma }}\text{GP}(m_{1},\theta_{1})+\frac{m_{2}^{\gamma }}{m_{1}^{\gamma }+m_{2}^{\gamma }}\text{GP}(m_{2},\theta_{2}),$$

and the perceived orientation is given by

2.3 $$\hat{\theta }=\tan^{-1}\left(\frac{m_{2}^{\gamma +1}-m_{1}^{\gamma +1}}{m_{2}^{\gamma +1}+m_{1}^{\gamma +1}}\tan\frac{\theta_{2}-\theta_{1}}{2}\right)+\frac{\theta_{2}+\theta_{1}}{2}.$$

This is the first model of the five nested models in our previous study [7]. Similar to the prediction of perceived phase [7], the power summation model [5] and the two-stage model [12] give the same prediction of the perceived orientation as given by equation (2.3).

*Model 2: contrast-weighted summation plus contrast gain-enhancement.* This is the fourth nested model in our previous study [7] with gain-control threshold = 0. The fused GP vector is given by

2.4 $$\text{GP}(\hat{m},\hat{\theta })=\frac{m_{1}^{\gamma }}{m_{1}^{\gamma }+m_{2}^{\gamma }}\left(1+{\left(\frac{m_{2}}{g_{e}}\right)}^{\gamma }\right)\text{GP}(m_{1},\theta_{1})+\frac{m_{2}^{\gamma }}{m_{1}^{\gamma }+m_{2}^{\gamma }}\left(1+{\left(\frac{m_{1}}{g_{e}}\right)}^{\gamma }\right)\text{GP}(m_{2},\theta_{2}).$$

The formula for the perceived orientation is given in electronic supplementary material.

*Model 3: DSKL model.* The DSKL model consists of three layers for each eye before the binocular linear summation site: (i) a selective signal layer that receives both gain-control and gain-enhancement from the other eye and outputs the signal to the binocular summation site; (ii) a non-selective gain-control layer that first extracts and sums image contrast energy (ε) across frequency channels and orientations (total contrast energy, TCE) and then exerts gain-control to the other eye's three layers separately with different gain-control efficiencies; (iii) a gain-enhancement layer that extracts image contrast energy ($\varepsilon^{*}$) (TCE*) and exerts gain-enhancement only to the other eye's signal layer. For normal vision without eye bias, the full DSKL model has five parameters: gain-control threshold *g*_c_, gain-enhancement threshold *g*_e_, the threshold for gain-control of gain-control *g*_cc_, the threshold for gain-control of gain-enhancement *g*_ce_, and the gamma exponent *γ* for calculation of image contrast energy. When *g*_c_ = 0 and *g*_cc_ = 0, the fused GP vector is given by

2.5 $$\text{GP}(\hat{m},\hat{\theta })=\frac{m_{1}^{\gamma }}{m_{1}^{\gamma }+m_{2}^{\gamma }}\left(1+\frac{{\left(\frac{m_{2}}{g_{e}}\right)}^{\gamma }}{1+{\left(\frac{m_{1}}{g_{\mathrm{ce}}}\right)}^{\gamma }}\right)\text{GP}(m_{1},\theta_{1})+\frac{m_{2}^{\gamma }}{m_{1}^{\gamma }+m_{2}^{\gamma }}\left(1+\frac{{\left(\frac{m_{1}}{g_{e}}\right)}^{\gamma }}{1+{\left(\frac{m_{2}}{g_{\mathrm{ce}}}\right)}^{\gamma }}\right)\text{GP}(m_{2},\theta_{2}).$$

The formula for the perceived orientation is given in the electronic supplementary material.

## 3. Results

In order to explore how the orientation information is combined to form a unified fused percept, we varied both the interocular (dichoptic) and monocular orientation difference and the interocular and monocular contrast ratio. Varying the interocular contrast ratio has provided strong constraints and important insights into both phase and contrast combination [6,7,14].

For each dichoptic orientation combination, one eye was stimulated with a fixed ‘base’ contrast (10%, 20%, 40% or 60%), whereas the fellow eye was presented with one of the four contrast levels, resulting in eight interocular contrast ratios varying from 0.25 to 4 for each orientation combination (see Material and methods). For example, when one eye was stimulated with a 20% base contrast, the fellow eye was presented with 5% (which is near the monocular threshold of contrast detection, see Methods), 10%, 15% or 20% contrast. We also used identical conditions to compare between binocular and monocular combination of orientation.

**Figure 2.**
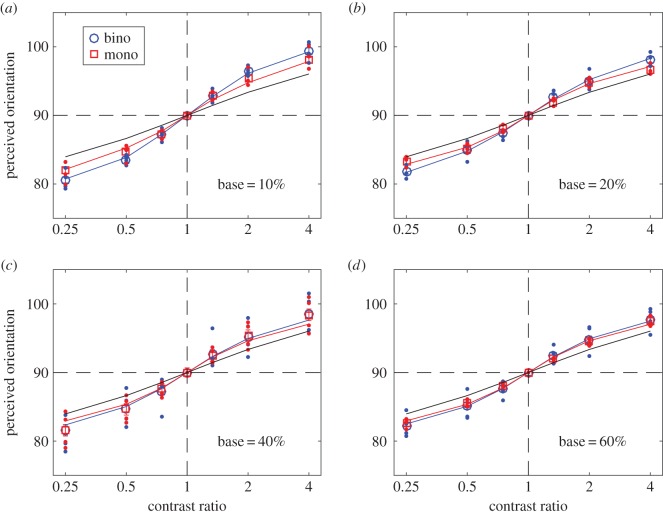
Perceived orientation when the orientation difference of the two input gratings was 20 degrees. The red and blue curves are the best fits of the DSKL model [7] to the data (red squares and blue circles) averaged across four observers, and the black line is the prediction of orientation linear vector summation model. The red and blue dots indicate data for individual observers. Error bars: ± 1 s.e.

**Figure 3.**
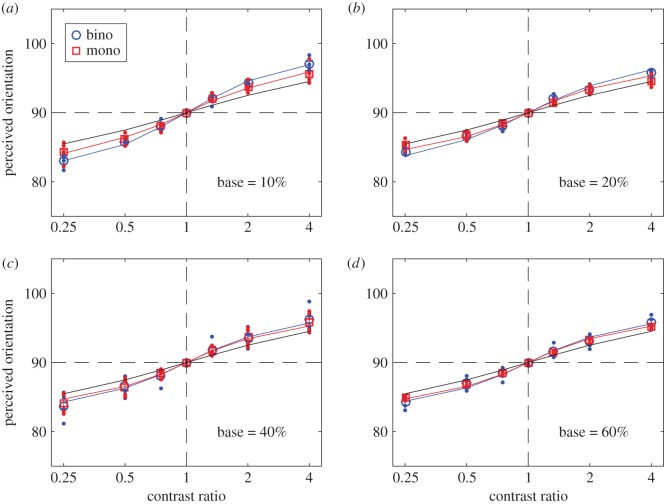
Perceived orientation when the orientation difference of the two input gratings was 15 degrees. The red and blue curves are the best fits of the DSKL model [7] to the data (red squares and blue circles) averaged across four observers, and the black curve is the prediction of orientation linear vector summation model. The red and blue dots indicate data for individual observers. Error bars: ± 1 s.e.

**Figure 4.**
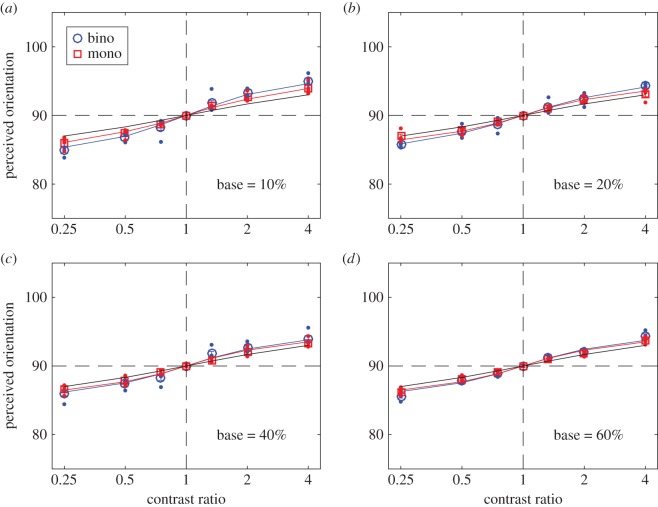
Perceived orientation when the orientation difference of the two input gratings was 10 degrees. Error bars: ± 1 s.e.

**Figure 5.**
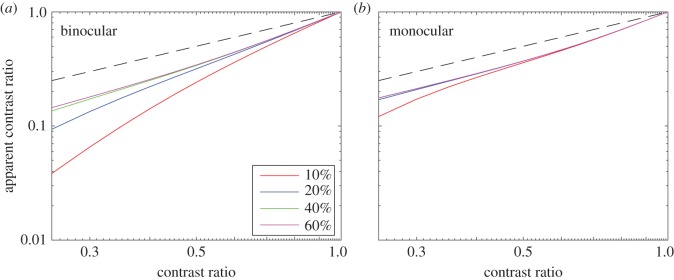
Apparent contrast ratio as a function of the stimulus contrast ratio when the base contrast was fixed at 10% (red), 20% (blue), 40% (green) and 60% (magenta). Dashed black line shows the prediction of linear summation, for which the apparent contrast ratio is identical to the contrast ratio.

At each base contrast and contrast ratio, the raw rating data were analysed using SDT to calculate the perceived orientation when a grating with one orientation was presented to the left eye and a grating with a different orientation was presented to the right eye (binocular orientation combination), or when the two gratings were presented in the same eye (monocular orientation combination). In order to remove any possible bias, for each observer, the results were averaged across eyes and directions of rotation. These results were averaged across the four observers and were fitted with several orientation combination models (figures 2–5). We also fitted the individual data (shown in electronic supplementary material).

Figure 2*a* shows the results for an orientation difference of 20 degrees and a base contrast of 10%. For both binocular (blue) and monocular (red) conditions, perceived orientation shifted from the left eye's orientation (80 degrees) to the right eye's orientation (100 degrees) as the right eye/left eye contrast ratio increased. Open circles are the average data, small dots show the individual data. The red and blue curves are the best fits of an orientation combination model, i.e. the DSKL model [7] (see Material and methods), to the data averaged across four observers, and the black line is the prediction of a linear vector summation model. The data fall close to the linear summation prediction when the interocular contrast ratio is close to 1; however, when the two input orientations had different contrasts (contrast ratio ≠ 1), the data shifts away from the orientation predicted by linear summation, and towards the orientation with the higher contrast. This shift is a consequence of mutual suppression between the two gratings. As base contrast increases (figure 2*b*–*d*), the mutual suppression decreases, resulting in a smaller deviation from the linear prediction. This reduced mutual suppression can be explained by mutual enhancement between the two gratings. It is also noteworthy that while monocular and binocular combination followed very similar patterns, both mutual suppression and mutual enhancement were systematically stronger under binocular than under monocular conditions.

Both mutual suppression and enhancement were independent of input orientation difference over the limited range tested. Similar results were obtained when the input orientation difference was 15 (figure 3) or 10 degrees (figure 4). The astute reader might note that the two models converge as the orientation difference becomes smaller at all base contrast levels (compare figures 2 and 4).

Figure 5 summarizes our main results by comparing the apparent contrast ratio (i.e. the ratio of the monocular model outputs of the two eyes, see §2.8) to the input stimulus contrast ratio. Without any interorientation interaction, the apparent contrast ratio is identical to the contrast ratio (black dashed lines, which show the prediction of linear summation in figure 5). Mutual suppression reduced the apparent contrast ratio when the stimulus contrast ratio was below 1. Note that this reduction in the apparent contrast ratio was substantially greater under binocular (figure 5*a*) than under monocular (figure 5*b*) conditions. Averaged across the four observers, figure 5 demonstrates:

(1) Mutual suppression under both binocular and monocular conditions—i.e. the apparent contrast is less than the input stimulus contrast.
(2) Mutual enhancement under both binocular and monocular conditions—i.e. as base contrast increases (shown by the different coloured curves in figure 5*a,b*), the mutual suppression decreases, resulting in a smaller deviation from the linear prediction.
(3) Both mutual suppression and enhancement are stronger under binocular conditions than under monocular conditions.

We note that there are substantial individual differences among observers (see electronic supplementary material and discussion below). The variance across observers is summarized in electronic supplementary material, table S1. We note that, for observers D.V. and O.E., there is a strong effect of mutual suppression and mutual enhancement under binocular conditions, whereas under monocular conditions, mutual suppression is a weak effect, and there is no mutual enhancement. For these two observers, the monocular and dichoptic data are significantly different (*p* < 0.05) at 10% base contrast; the mutual suppression was much stronger under binocular conditions than under monocular conditions. However, as base contrast increased, mutual enhancement cancelled the mutual suppression under binocular conditions, resulting in similar data to those under monocular conditions at high base contrast (60%). For observers Y.S. and S.T., the monocular and dichoptic data are very similar; under both binocular and monocular conditions, observer Y.S. showed strong suppression and enhancement, whereas observer S.T. showed strong suppression but no enhancement.

### 3.1. Model fitting

Table 4 shows model parameters when fitting the DSKL model to the average data of four observers (red and blue curves in figures 2–4). We compared three nested models, in which a previous model is nested within its successor. When the threshold of gain-control of gain-enhancement *g*_ce_ = *∞*, the DSKL model is simplified to be model 2, and when the gain-enhancement threshold *g*_e_ = *∞*, model 2 is further simplified to be model 1. Their fitting statistics (reduced Chi-square (*χ*^2^/*v*) and corrected Akaike information criterion (AIC—see electronic supplementary material for details) are shown in tables 5 and 6.

**Table 4.** DSKL model parameters. The standard errors were estimated from the standard deviation of the residuals and the Jacobean matrix at the point of best fit in parameter space.

	*g*_c_	*g*_cc_	*g*_e_	*g*_ce_	γ
bino	0	0	0.053 ± 0.009	0.043 ± 0.006	2.47 ± 0.54
mono	0	0	0.022 ± 0.003	0.020 ± 0.004	3.57 ± 1.18

**Table 5.** Model fitting statistics (binocular). *N*_p_, the number of parameters; *v*, the number of the degrees of freedom; AIC_c_, the Akaike information criterion with a correction for finite sample sizes.

			D.V.		Y.S.		S.T.		O.E.		average				
	*N_p_*	*v*	*χ*^2^	*χ*^2^*/v*	*χ*^2^	*χ*^2^*/v*	*χ*^2^	*χ^2^/v*	*χ*^2^	*χ*^2^*/v*	*χ*^2^	*χ*^2^*/v*	AIC_c_	*F*	*p*(*F*)
model 1	**1**	35	41.3	1.18	108.2	3.09	45.1	1.29	57.6	1.65	55.7	1.59	57.8		
model 2	**2**	34	22.9	0.67	41.3	1.22	45.1	1.33	57.6	1.69	33.8	0.99	38.2	22.0	<0.001
DSKL	**3**	33	20.4	0.62	41.2	1.25	45.1	1.37	36.7	1.11	25.7	0.78	32.5	10.4	<0.001

**Table 6.** Model fitting statistics (monocular). *N*_p_, the number of parameters; *v*, the number of the degrees of freedom; AIC_c_, the Akaike information criterion with a correction for finite sample sizes.

			D.V.		Y.S.		S.T.		O.E.		Average				
	*N_p_*	*v*	*χ*^2^	*χ*^2^*/v*	*χ*^2^	*χ*^2^*/v*	*χ*^2^	*χ*^2^*/v*	*χ*^2^	*χ*^2^*/v*	*χ*^2^	*χ*^2^*/v*	AIC*_c_*	*F*	*p*(*F*)
model 1	**1**	35	35.7	1.02	49.9	1.43	94.7	2.71	42.9	1.23	42.3	1.21	44.4		
model 2	**2**	34	35.7	1.05	35.2	1.03	94.7	2.79	43.1	1.27	38.0	1.12	42.4	3.85	<0.001
DSKL	**3**	33	35.7	1.08	25.8	0.78	94.7	2.87	43.0	1.30	29.2	0.89	36.0	9.95	<0.001

The comparison of two neighbouring models was made through an *F*-test (see electronic supplementary materials for details) with the *F*-value given in the row of the second model (*F*-test and its *p*-value are only shown for the average data). When fitting averaged data, under both binocular and monocular conditions, the *F*-test shows that the goodness of fit was significantly improved by adding interocular gain-enhancement to the model (model 2 versus model 1). Although the *F*-test also shows a further improvement of fit performance by adding gain-control of gain-enhancement (DSKL model versus model 2), we cannot exclude the possibility of overfitting the model to the noise in the data, because the reduced Chi-square (*χ*^2^/*v*) was less than one [44]. In this study, we did not have enough data to test gain-control of gain-enhancement in the DSKL model, although it significantly improved the model fit in our previous studies [7,45]. For three observers, adding gain-enhancement (observers D.V. and Y.S.) and further adding gain-control of gain-enhancement (observer O.E.) also improved the fits to the individual data under binocular conditions (table 5). However, for the fourth observer (S.T.), whose data showed no interocular gain-enhancement (the interocular suppression remained constant when base contrast increased—see electronic supplementary material), neither adding gain-enhancement nor adding gain-control of gain-enhancement (model 2 or DSKL model) improved the fit. Under monocular conditions (table 6), three observers showed no interocular gain-enhancement, and no improvement was observed when fitting their data with model 2 or the DSKL model.

## 4. Discussion

Earlier behavioural studies showed that orientation discrimination thresholds are lower under binocular than monocular conditions when the stimuli are brief and have low contrast, but the binocular advantage is essentially eliminated for contrasts above about 15% [17]. However, our aim was not to compare monocular and binocular sensitivity, but rather to directly investigate the mechanism behind the combined fused percept that substitutes the two separate monocular percepts. Our results show that stimuli that differ in orientation by up to 20 degrees around the vertical meridian, within the ‘effective limit on single vision’ [35] are combined to form a single percept, regardless of whether the two orientations are presented monocularly or binocularly, with a bias that depends on the interocular contrast ratio, demonstrating that the brain can produce a coherent percept from a combination of two different orientations that is similar to a pure single-orientation stimulation.

There is no previous evidence on how different orientations presented to the two eyes are combined when the task is to explicitly address the perceived orientation and not to use it as a cue for depth (we note that in our experiments using small GPs and brief exposures, slant was not experienced by any of the participants (or by the authors)). Because both physiology and behaviour showed orientation tuning of about ±10 degrees around the preferred orientation, we assume that dichoptic stimuli, with a total orientation difference of up to 20 degrees used here, probably activate the same processing channel with the same orientation preference. Importantly, a substantial proportion of cells in both cat striate cortex [32] and macaque V1 [33] differ significantly in the preferred orientations of the two eyes. In the same vein, it has been suggested that if two gratings that activate the same processing channel with the same orientation preference are presented monocularly, they provide a stable percept of a single orientation [40].

Based on recordings from neurons in the cat striate cortex, Hubel and Wiesel showed that the inputs from the two eyes exhibited linear spatial summation [46,47]. Smith *et al.* [48] showed in monkey simple cells that the effectiveness of a stimulus in producing a response reflects interocular differences in the relative balance of inputs to a given cell; however, the eye of origin has no specific consequence. Simple cells showed linear spatial summation between the left- and right-eye receptive fields. The same mechanism of linear summation has been suggested to account for orientation selectivity, before a cell's nonlinear mechanisms. This suggestion appears to be applicable to our findings when the interocular contrast ratio is close to 1. However, as shown in figures 2–4, the results depart from simple linear (vector) summation when the two eyes have different contrasts. This nonlinear contrast influence on interocular effects is reminiscent of the nonlinear contrast influence on context effects of collinearly oriented flanking elements falling outside target's receptive field [49,50]. Based on our DSKL model, the departures from linearity, both under monocular and dichoptic viewing, may be explained on the basis of mutual suppression and mutual enhancement, both of which are stronger under dichoptic than monocular conditions. So far as we know, no physiological studies have directly addressed the mutual interaction of two gratings with similar orientations. However, based on studies using gratings with orthogonal orientations, previous physiological studies [51,52] demonstrated that interocular suppression is substantially different from monocular cross-orientation suppression. These studies suggest that interocular suppression is mediated by inhibitory circuitry within the visual cortex, whereas monocular cross-orientation suppression is mediated by subcortical mechanisms.

We fitted several models to the data. The linear vector summation model (black lines in figures 2–4) is clearly a poor fit when the interocular contrast ratio is not equal to one. The data are well fitted by a gain-control model (model 2) and by the full DSKL model [7]—red and blue curves in figures 2–4—which has been used to successfully predict binocular combination of perceived phase and contrast [7] and also contrast discrimination [45,53]. Here, we show that it can also predict both the binocular and monocular combination of nearby orientations.

Binocular orientation and phase combination differ from each other in several important ways. In binocular phase combination, the interocular suppression becomes stronger as the base contrast increases, and at low contrast and short stimulus duration, the summation is almost linear, without interocular suppression [27]. However, in binocular orientation combination, the interocular suppression becomes weaker as the base contrast increases. We assume interocular enhancement to account for this decreasing interocular suppression at higher contrasts. This interocular enhancement cannot be observed directly in binocular phase combination because of the strong mutual interocular suppression in observers with normal vision. However, it can be observed in observers with amblyopia, because of the weak or absent interocular suppression from the non-dominant eye to the dominant eye [45].

It is not altogether surprising that the binocular orientation and phase combinations have different behaviours given the differences in stimuli and tasks. For example, stimuli differing in phase can be combined into one mathematically by simply summing the two stimuli. However, stimuli differing in orientation cannot be combined into one mathematically by simply summing the two. Moreover, in the phase task, observers match the perceived phase of the dichoptically combined gratings to a binocularly viewed reference line, whereas in the orientation task, observers match the perceived orientation to an internal subjective standard.

Our study was limited to briefly presented stimuli with orientation differences of up to 20 degrees. We have not explored larger orientation differences, where the two orientations cannot be combined into a percept of a single orientation, and are probably processed by separate channels [39]. Similarly, we have not explored long durations, where two Gabor stimuli of slightly different orientations presented binocularly will continue to produce a single fused percept, but temporally interleaved monocular stimuli do not, instead producing an oscillating percept, most likely owing to after-images [42]. For our stimuli, it is plausible that the two inputs are processed by a single channel, because the orientation differences are small, within Braddick's [35] ‘effective limit on single vision’. Of course, this suggestion merits verification in an experiment on a cellular level with a similar set of stimuli.

## Supplementary Material

Supplemental Material

## Data accessibility

Data files relevant to this study can be found in the Dryad database: http://dx.doi.org/10.5061/dryad.4q730 [54].
