# Supplementary material for: Binocular combination of stimulus orientation
Source: R Soc Open Sci. 2016 Nov 16;3(11):160534. doi: 10.1098/rsos.160534 (PMC5180139; doi:10.1098/rsos.160534)
Supplement: Supplemental Material [file rsos160534supp1.pdf]

Supplemental material

Model prediction of perceived orientation

When the two eyes are presented with two Gabor patches (GPs) with slightly different orientations, a single cyclopean GP is perceived. A GP of one orientation is represented by a vector $\boldsymbol{GP}(m, \theta)$, with the vector length representing the GP's contrast m, and the vector angle representing the orientation angle θ. Let vectors $\boldsymbol{GP}(m_1, \theta_1)$ and $\boldsymbol{GP}(m_2, \theta_2)$ be two GPs presented to the two eyes, respectively. We assume that the cyclopean GP vector is given by a weighted vector summation, i.e.,

$$\boldsymbol{GP}(\widehat{m}, \hat{\theta}) = w_1\boldsymbol{GP}(m_1, \theta_1) + w_2\boldsymbol{GP}(m_2, \theta_2). \quad \text{(S1)}$$

The perceived orientation $\hat{\theta}$ is given by,

$$\hat{\theta} = \tan^{-1}\left(\frac{w_2m_2 - w_1m_1}{w_2m_2 + w_1m_1}\tan\frac{\theta_2 - \theta_1}{2}\right) + \frac{\theta_2 + \theta_1}{2}\ . \quad \text{(S2)}$$

Linear vector summation model. When two eyes are equally weighted, *i.e.*, $w_1 = w_2 = 1$, we have the linear vector summation model.

Model 1: contrast-weighted summation model (Simplified Ding-Sperling model). The Ding-Sperling model can be simplified to be a contrast-weighted summation model when the gain-control threshold = 0. The two weights are given by

$$w_1 = \frac{m_1^\gamma}{m_1^\gamma + m_2^\gamma} \quad \text{and} \quad w_2 = \frac{m_2^\gamma}{m_1^\gamma + m_2^\gamma}\ . \quad \text{(S3)}$$

It can be easily confirmed that, using Eq. (S3), Eq. (S1) can be reduced to be Eq. (2) and Eq. (S2) can be reduced to be Eq. (3).

Model 2: contrast-weighted summation plus contrast gain enhancement. In Model 2, the two weights are given by

$$w_1 = \frac{m_1^\gamma}{m_1^\gamma + m_2^\gamma}\left(1 + \left(\frac{m_2}{g_e}\right)^\gamma\right) \quad \text{and} \quad w_2 = \frac{m_2^\gamma}{m_1^\gamma + m_2^\gamma}\left(1 + \left(\frac{m_1}{g_e}\right)^\gamma\right) \quad . \qquad \text{(S4)}$$

Using Eq. (S4), the perceived orientation can be calculated from Eq. (S2).

Model 3: DSKL model. In Model 3, the two weights are given by

$$w_1 = \frac{m_1^\gamma}{m_1^\gamma + m_2^\gamma}\left(1 + \frac{\left(\frac{m_2}{g_e}\right)^\gamma}{1+\left(\frac{m_1}{g_{ce}}\right)^\gamma}\right) \quad \text{and} \quad w_2 = \frac{m_2^\gamma}{m_1^\gamma + m_2^\gamma}\left(1 + \frac{\left(\frac{m_1}{g_e}\right)^\gamma}{1+\left(\frac{m_2}{g_{ce}}\right)^\gamma}\right) \quad . \qquad \text{(S5)}$$

Using Eq. (S5), the perceived orientation can be calculated from Eq. (S2).

F-test for comparison of nested models

Let χ^2 be the chi-square, N_p be the number of model parameters and N_{data} be the number of observed data points. We have the number of degrees of freedom $\nu = N_{data} - N_p$,. If Model a is nested within Model b, the F-test that examines whether Model b significantly improves data fitting is given by,

$$F_{a,b} = \frac{\frac{\chi^2(a)-\chi^2(b)}{\nu(a)-\nu(b)}}{\frac{\chi^2(b)}{\nu(b)}}, \qquad \text{(S6)}$$

which compares the variance between models a and b with the variance inside model b and has F distribution with $[\nu(a) - \nu(b),\ \nu(b)]$ degree of freedom. When the F value is large enough, Model a can be rejected at a small false-rejection probability p(F).

The AIC for comparison of different models

We used the Akaike Information Criterion (AIC), a measure of the relative goodness of fit of a statistical model developed by Akaike (1974), to compare different models. Let N_p be the number of model parameters and L_{Max} is the maximized value of the likelihood function for the estimated model, AIC is defined as $AIC = 2N_p - 2\ln L_{Max}$. Assuming that the errors are normally distributed and independent, after ignoring the constant term, AIC is given by

$$AIC = \chi^2 + 2N_p \,. \qquad \text{(S7)}$$

To give a greater penalty for additional parameters, Burnham and Anderson (2002) recommended the AIC with a correction for finite sample sizes (AICc), which is given by,

$$AICc = AIC + \frac{2N_p(N_p+1)}{N_{data}-N_p-1}, \qquad \text{(S8)}$$

where N_{data} is the number of observed data points.

Results of individual observers

To illustrate the variance across observers, we show the individual results and model fits in Figs. S1-S12. All observers had mutual suppression under both binocular and monocular conditions, with stronger mutual suppression under binocular than monocular conditions. Three of the four observers, D.V. (Figs S1-S3), Y.S. (Figs S4-S6) and O.E. (Figs S10-12), had mutual enhancement under binocular condition, while only one observer Y.S. (Figs S4-S6) had mutual enhancement under monocular condition. Observer S.T. (Figs S7-S9) had no mutual enhancement either under binocular or monocular conditions. However, averaged across observers (Figure 3), the mutual enhancement can be observed under both binocular and monocular conditions. Table S1 summarized the variance across observers. We noticed that, for Observers D.V. and O.E., under binocular condition, the mutual suppression and mutual enhancement show strong effect, while under monocular condition, the mutual

suppression shows weak effect and the mutual enhancement shows no effect. For these two observers, the monocular and dichoptic data are significantly different ($p<0.05$) at 10% base contrast; the mutual suppression was much stronger under binocular condition than under monocular condition. However, when base contrast increased, the mutual enhancement canceled the mutual suppression under binocular condition, resulting in similar data to those under monocular condition at high base contrast (60%). For Observers Y.S. and S.T., the monocular and dichoptic data are very similar; under either binocular or monocular condition, Observer Y.S. showed strong effect in both suppression and enhancement while Observer S.T showed strong effect in suppression but no effect in enhancement.

Table S1. Variance across observers

Observers	Monocular condition		Binocular condition	
	Suppression	Enhancement	Suppression	Enhancement
D.V.	+	0	++	++
Y.S.	++	++	++	++
S.T.	++	0	++	0
O.E.	+	0	++	++

'++' : strong effect; '+': weak effect; '0': no effect.

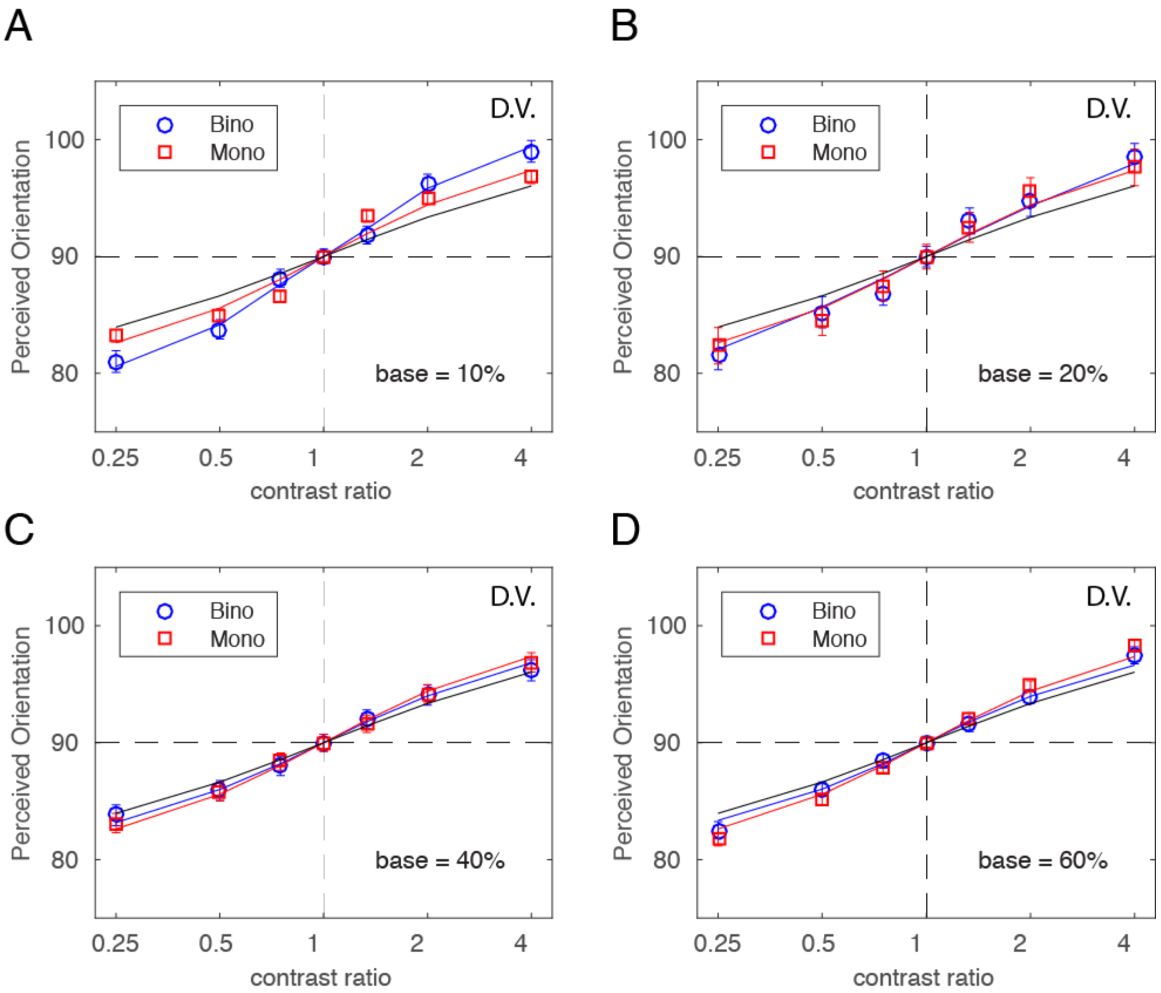

Figure S1. Perceived orientation when the orientation difference of the two input gratings was 20 degrees for Observer D.V. Under binocular condition (blue), the DSKL model is the best for fitting data to account for both interocular suppression and enhancement (see Table 4). However, under monocular condition, Model 1 is the best because no interocular enhancement was observed (see Table 5).

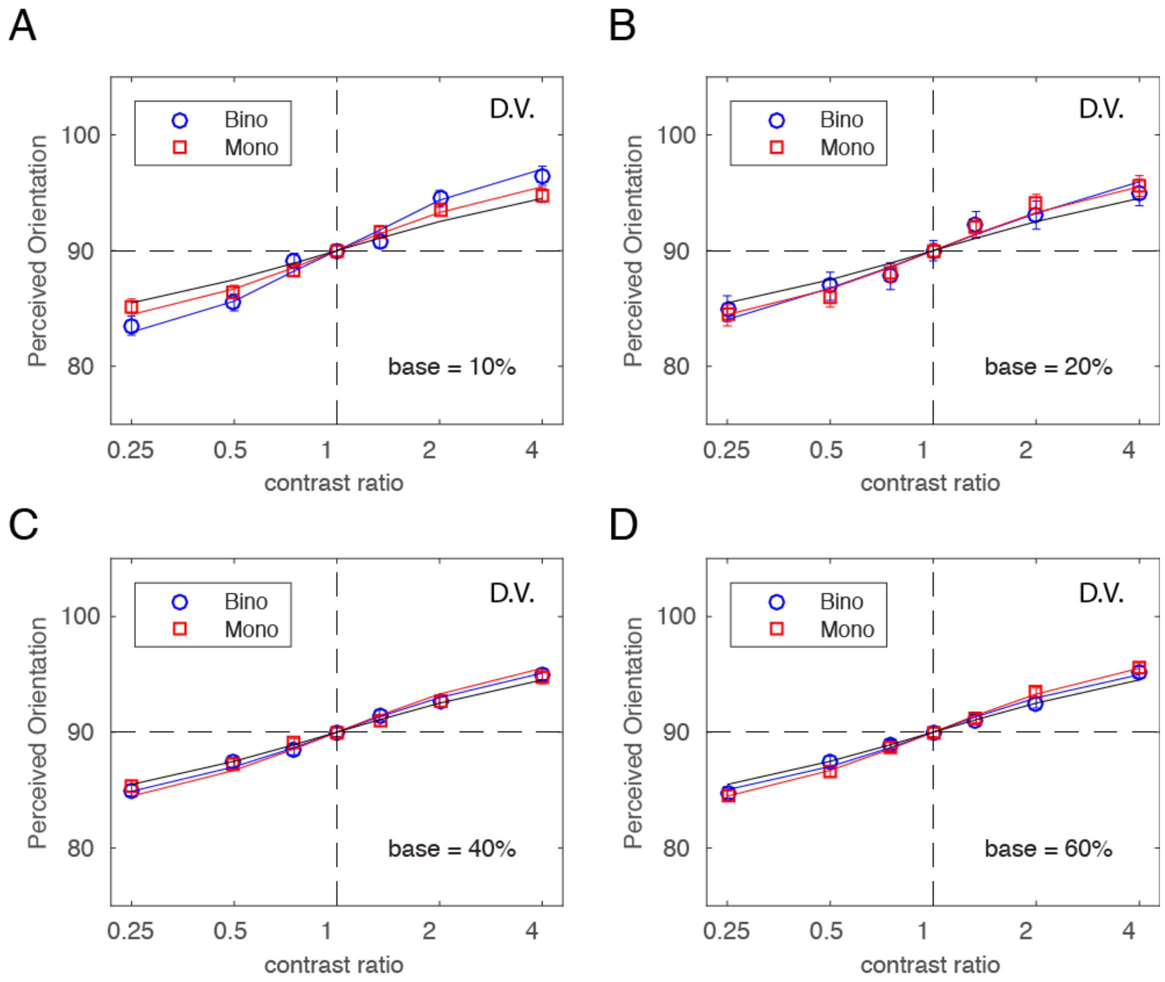

Figure S2. Perceived orientation when the orientation difference of the two input gratings was 15 degrees for Observer D.V.

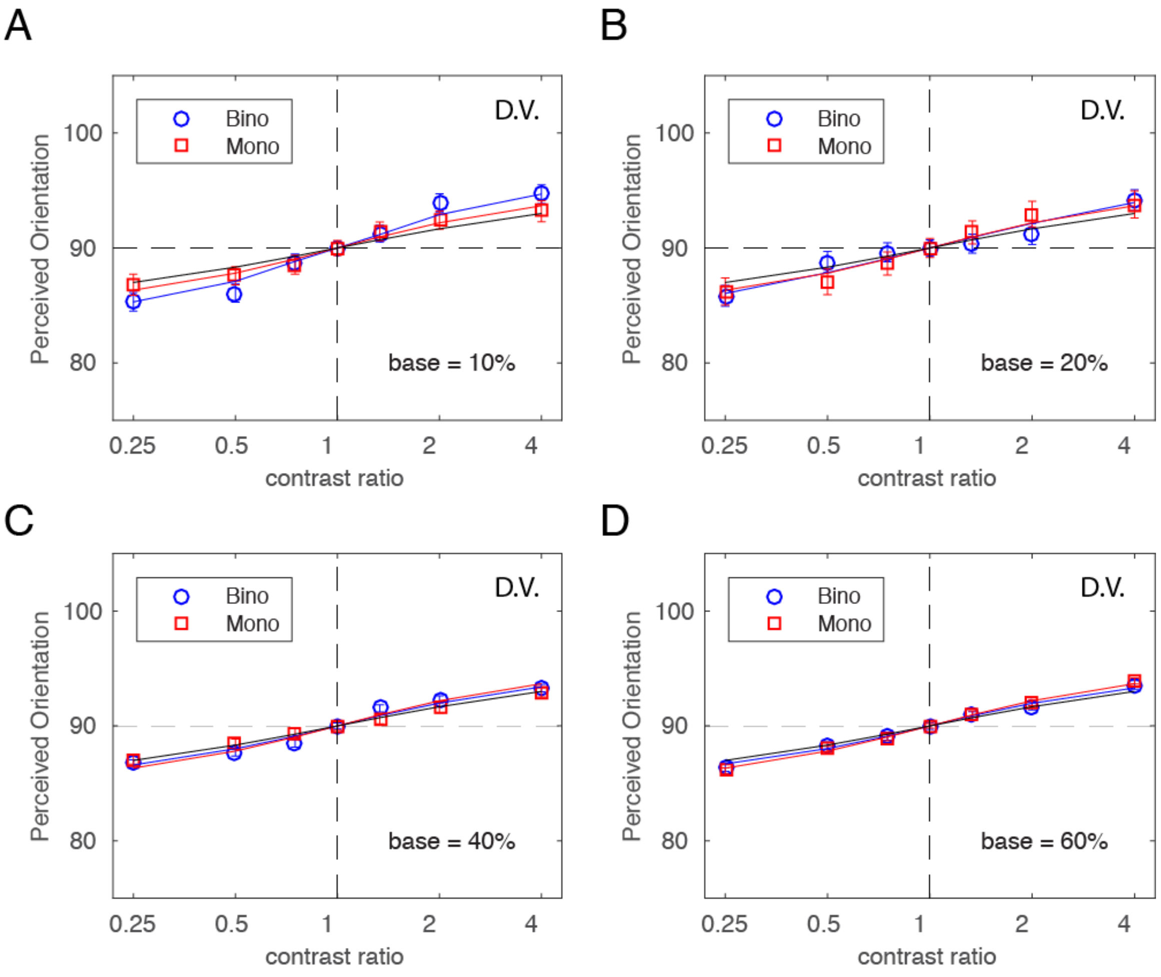

Figure S3. Perceived orientation when the orientation difference of the two input gratings was 10 degrees for Observer D.V.

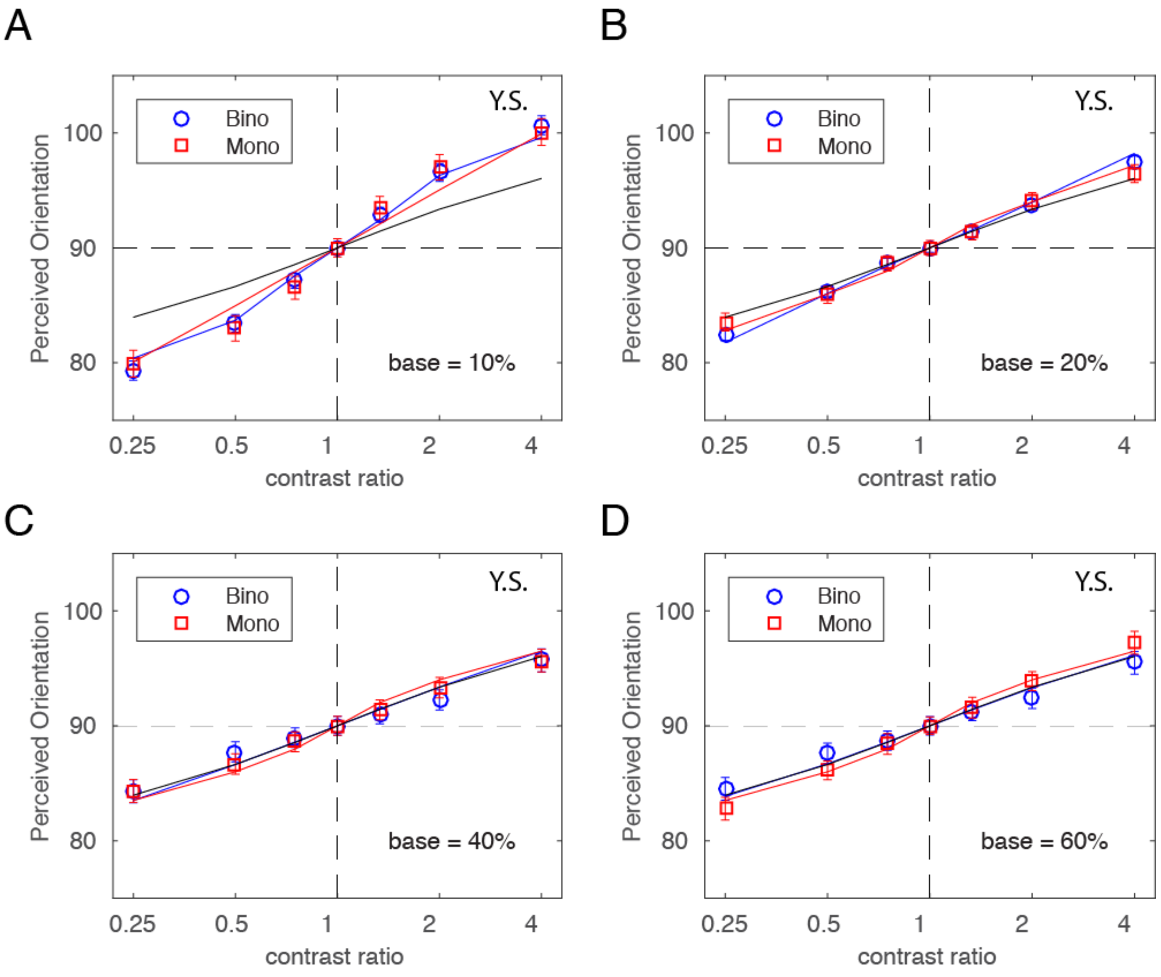

Figure S4. Perceived orientation when the orientation difference of the two input gratings was 20 degrees for Observer Y.S. Interocular enhancement was observed under both binocular (blue) and monocular (red) conditions. Model 2 is the best to account for both interocular suppression and enhancement under binocular condition (see Table 4) while the DSKL model is the best under monocular condition (see Table 5).

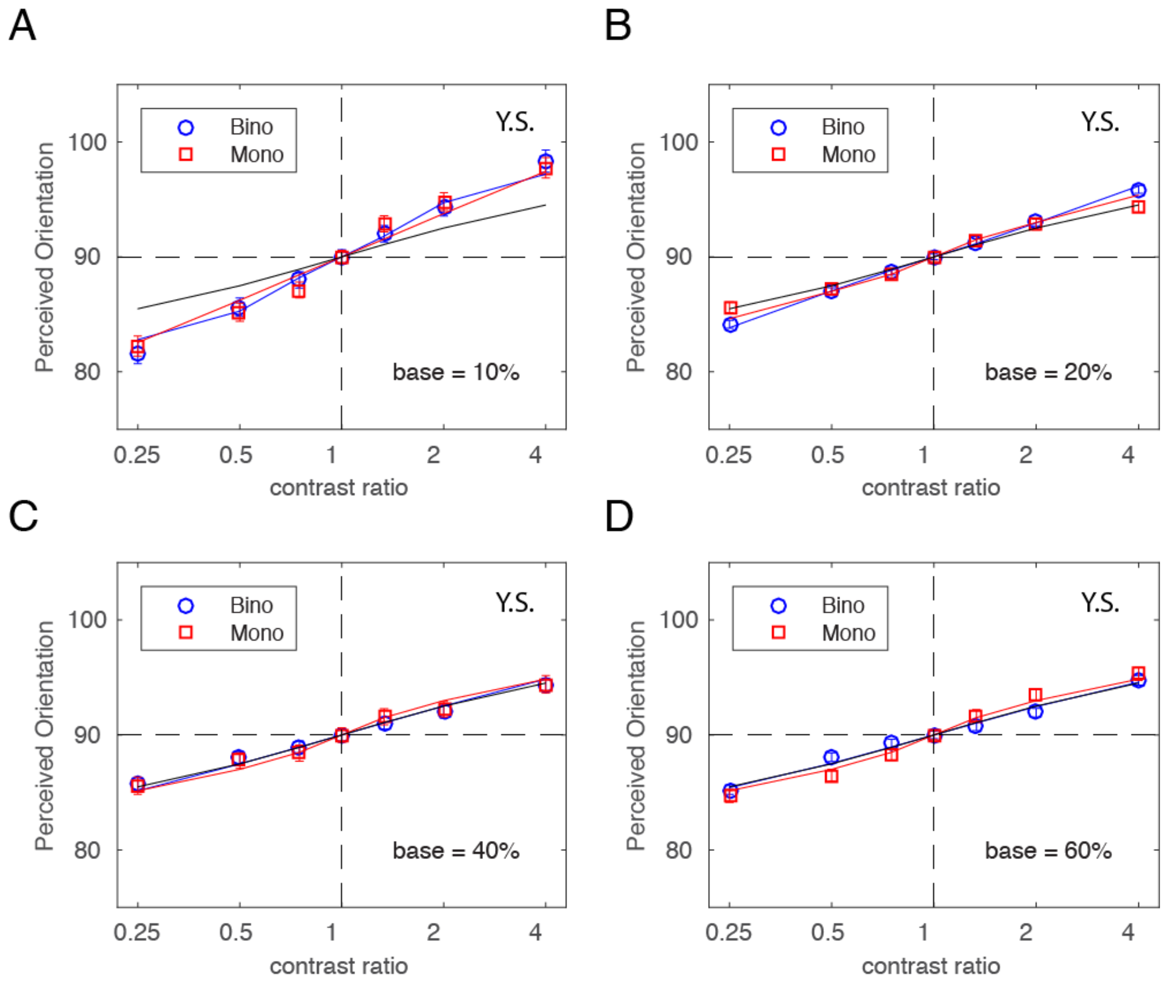

Figure S5. Perceived orientation when the orientation difference of the two input gratings was 15 degrees for Observer Y.S.

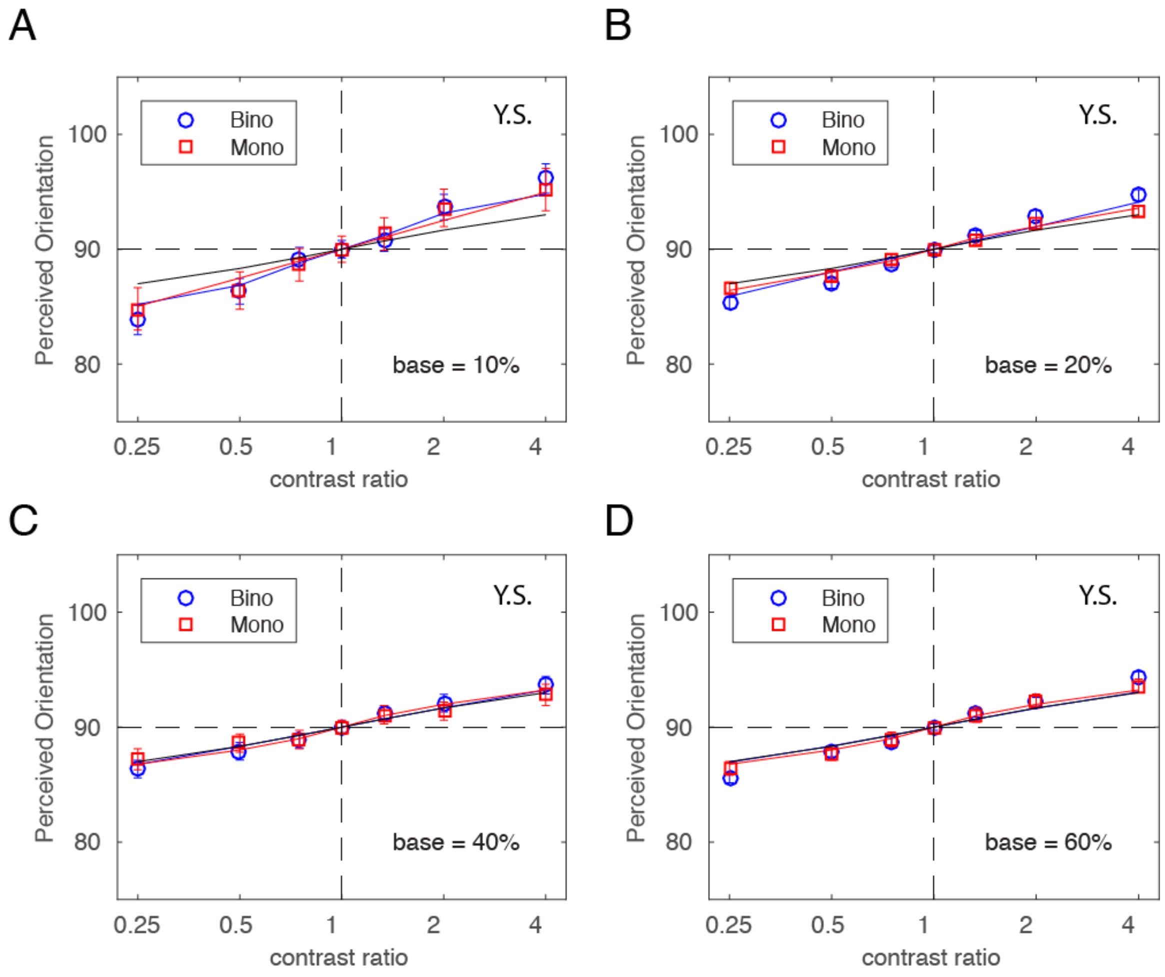

Figure S6. Perceived orientation when the orientation difference of the two input gratings was 10 degrees for Observer Y.S.

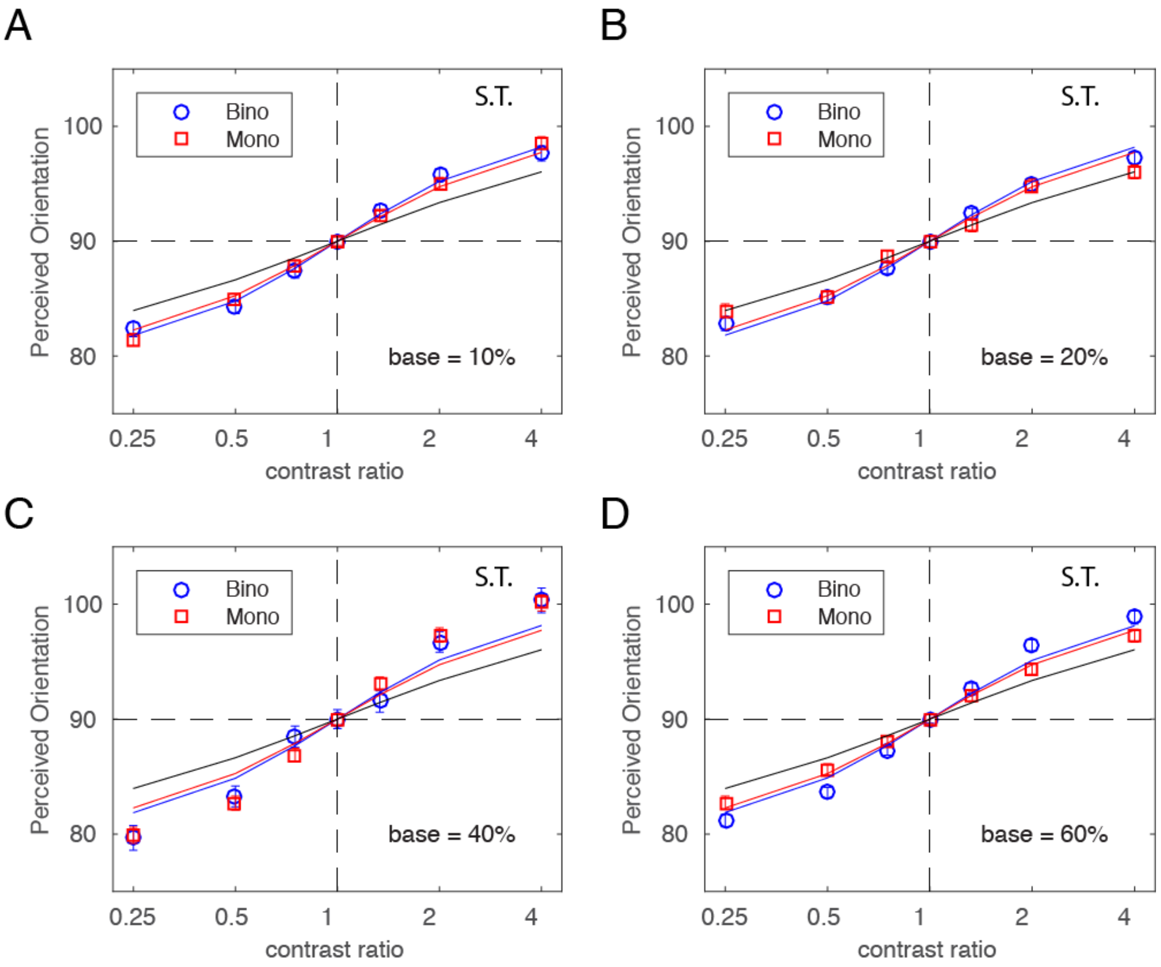

Figure S7. Perceived orientation when the orientation difference of the two input gratings was 20 degrees for Observer S.T. No interocular enhancement was observed either under binocular (blue) or monocular (red) conditions. Model 1 is best under both conditions (see Tables 4 and 5).

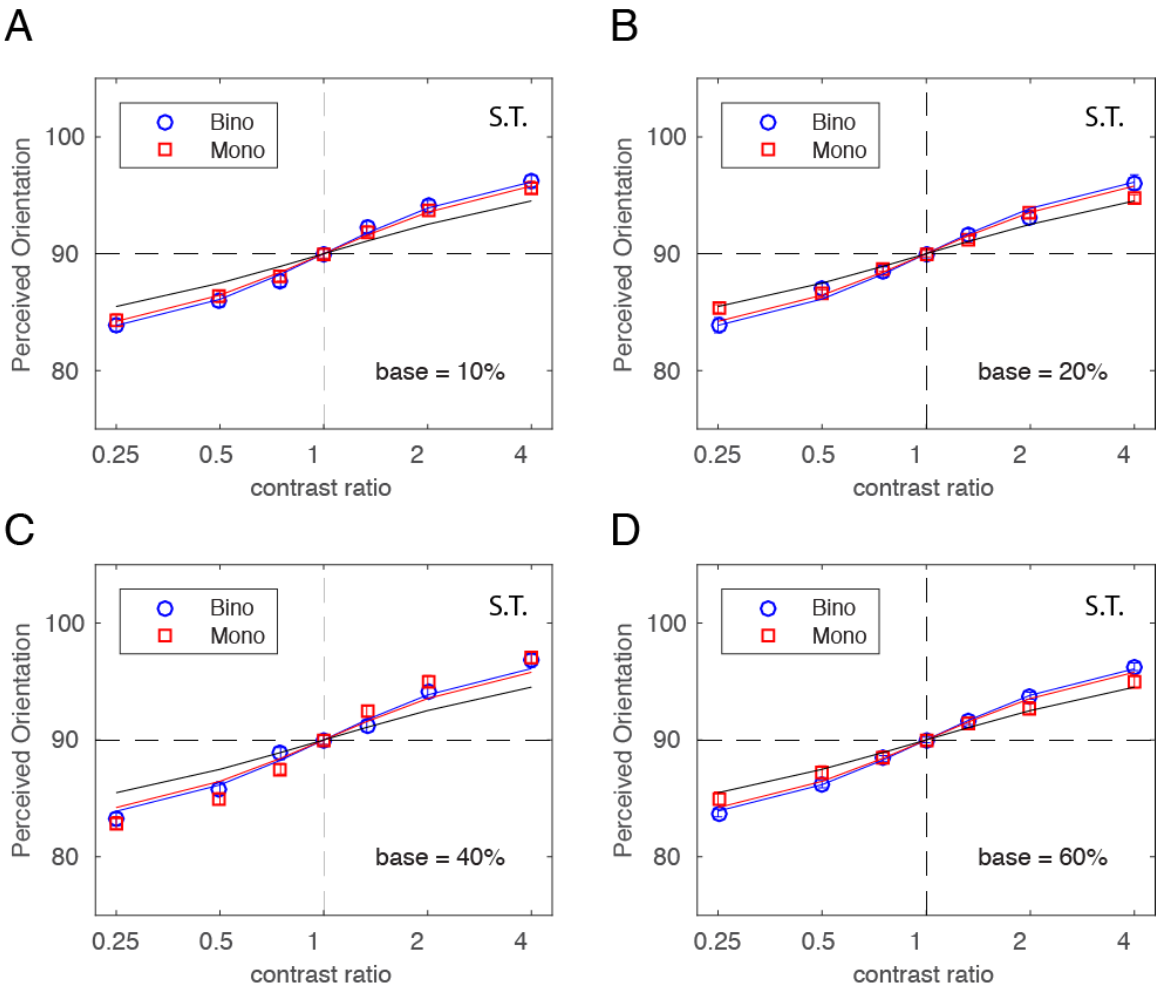

Figure S8. Perceived orientation when the orientation difference of the two input gratings was 15 degrees for Observer S.T.

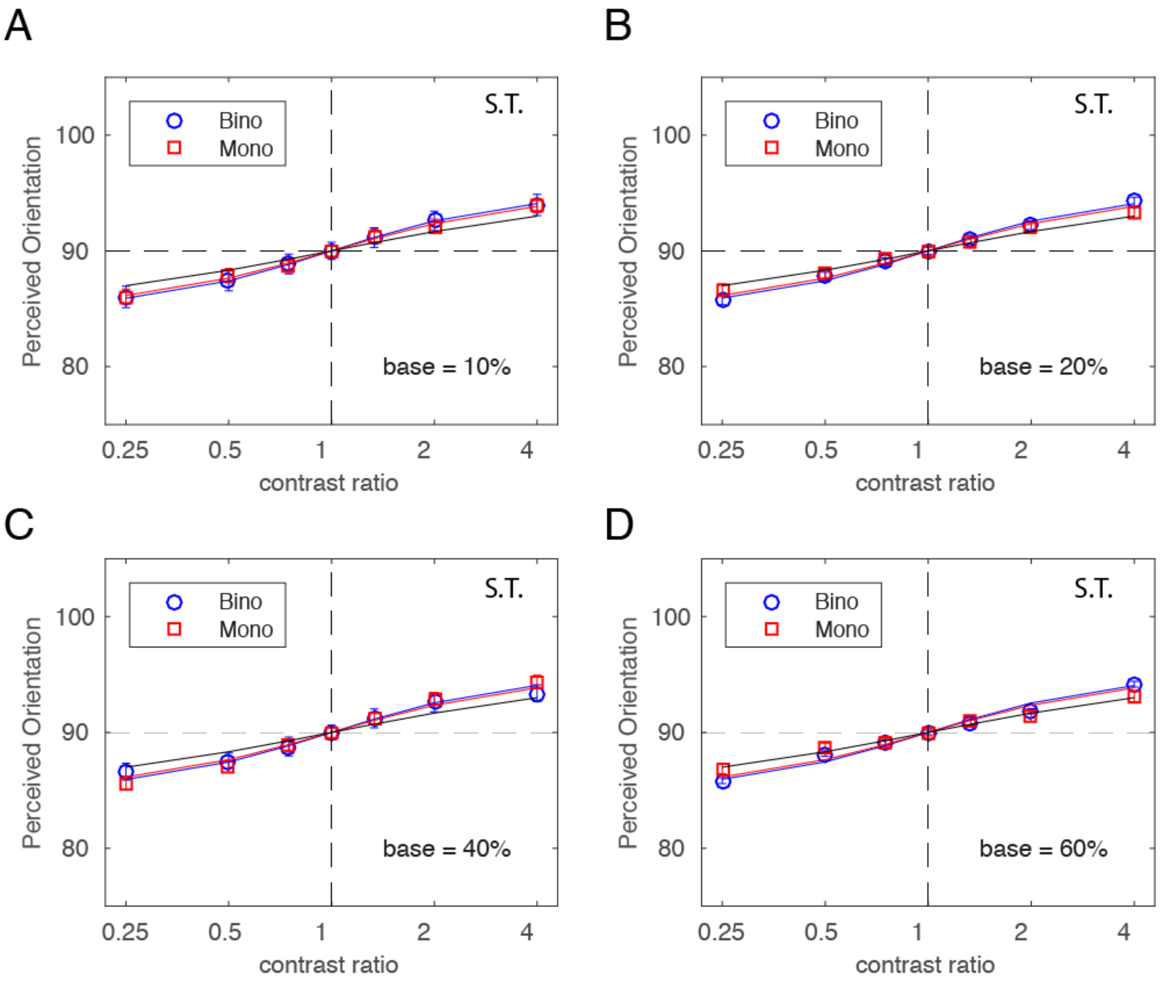

Figure S9. Perceived orientation when the orientation difference of the two input gratings was 10 degrees for Observer S.T.

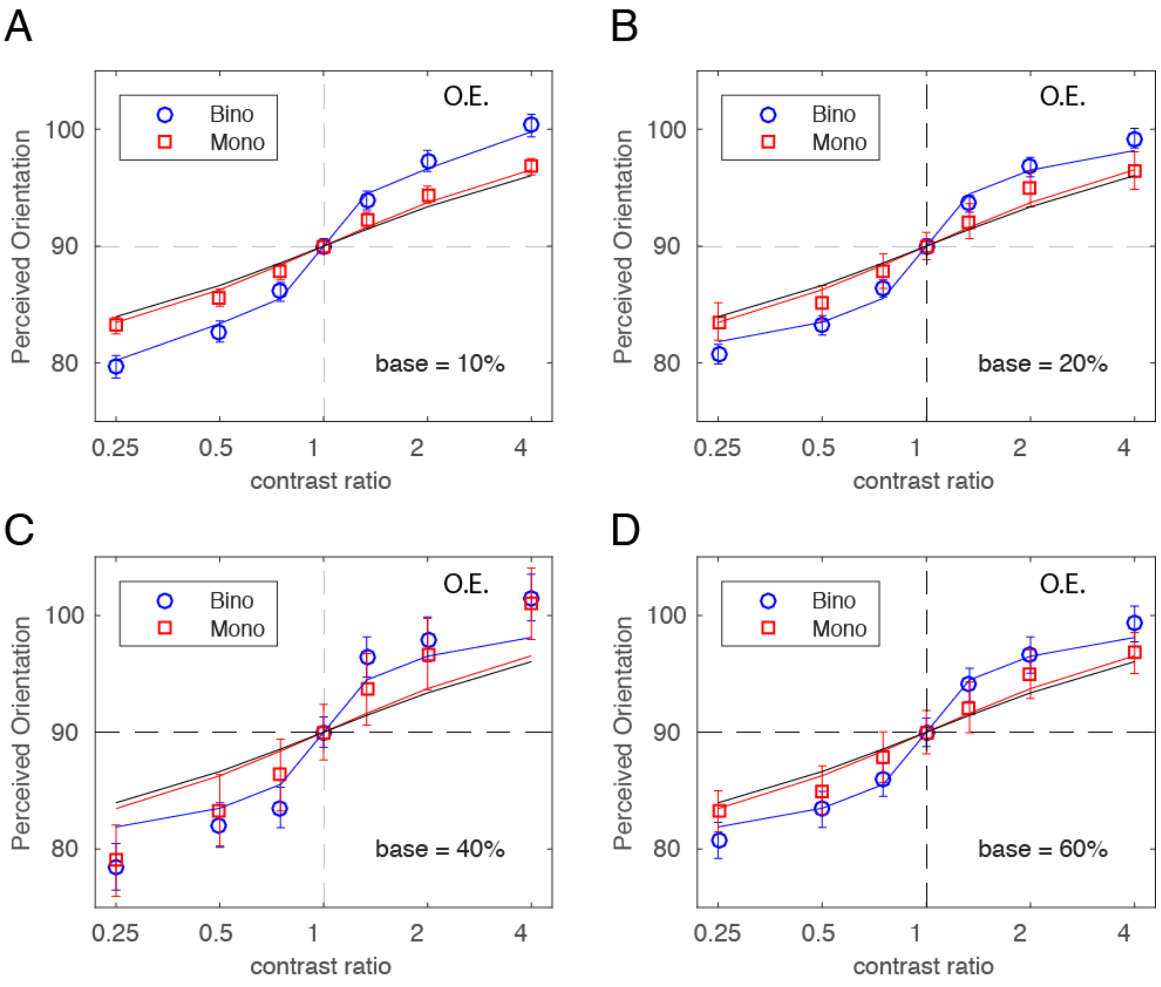

Figure S10. Perceived orientation when the orientation difference of the two input gratings was 20 degrees for Observer O.E. Under binocular condition (blue), the DSKL model is the best for fitting data to account for both interocular suppression and enhancement (see Table 4). However, under monocular condition, Model 1 is best because no interocular enhancement was observed (see Table 5).

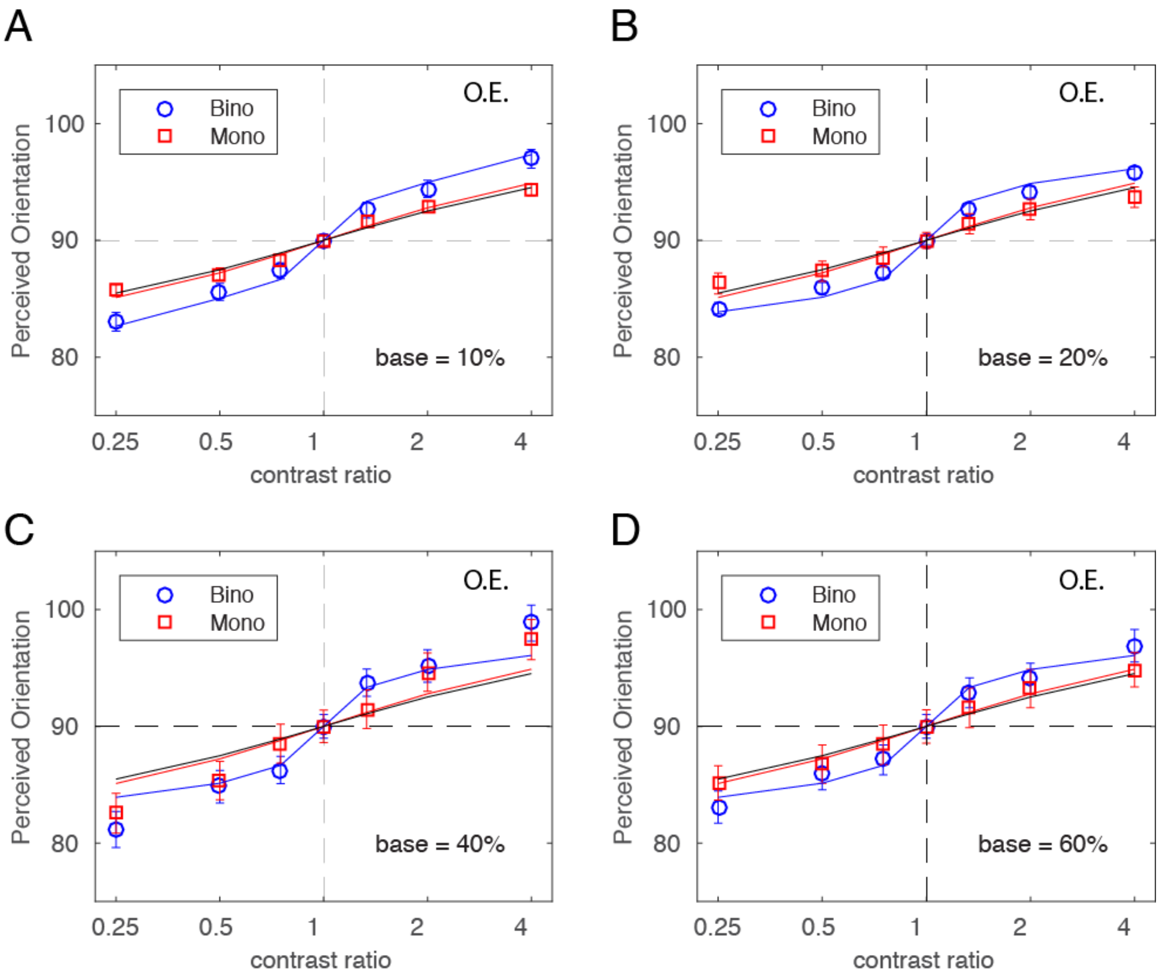

Figure S11. Perceived orientation when the orientation difference of the two input gratings was 15 degrees for Observer O.E

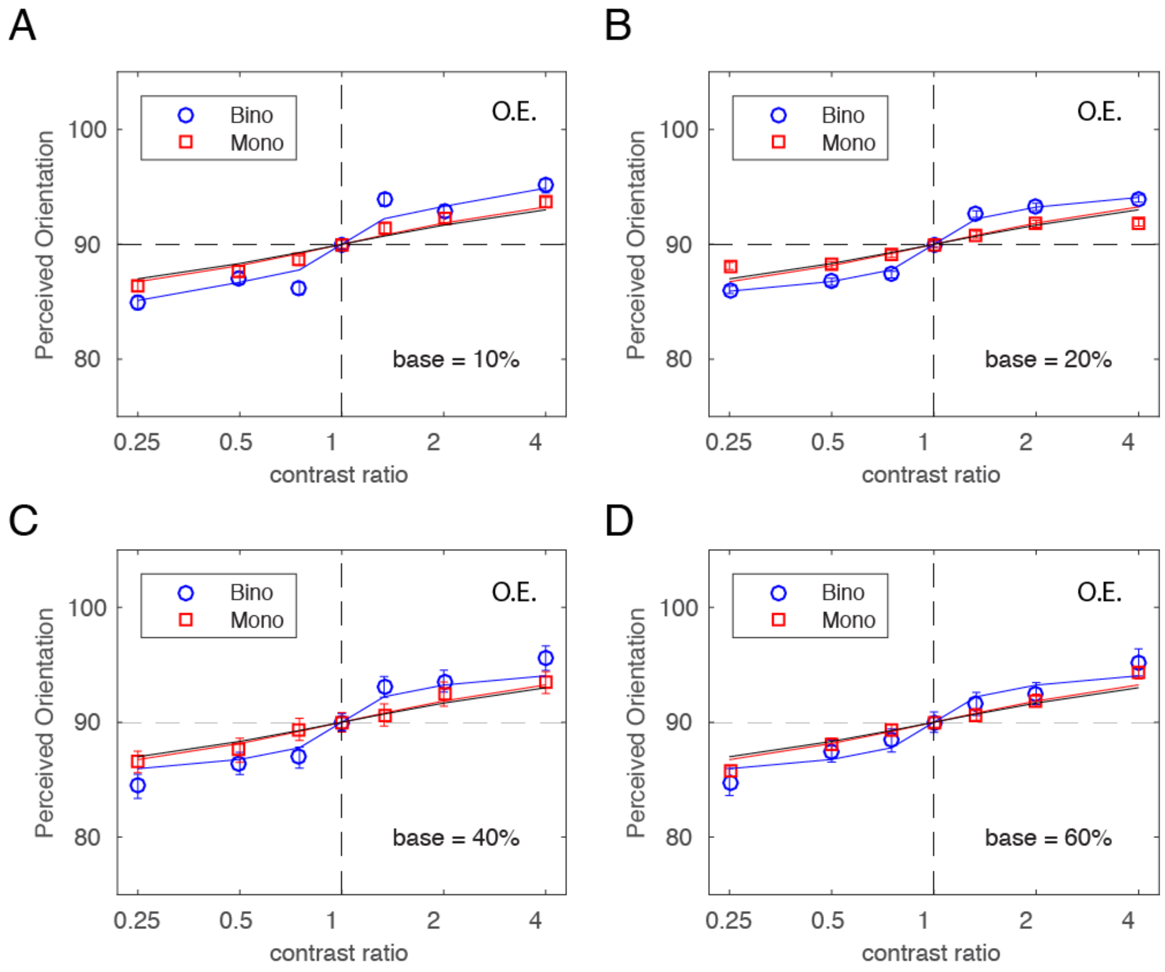

Figure S12. Perceived orientation when the orientation difference of the two input gratings was 10 degrees for Observer O.E

Akaike H (1974) A new look at the statistical model identification. Automatic Control, IEEE Transactions on 19:716-723.
Burnham KP, Anderson D (2002) Model selection and multi-model inference. A Pratical informatio-theoric approch Sringer.
